# Long-term blood glucose monitoring with implanted telemetry device in conscious and stress-free cynomolgus monkeys

**DOI:** 10.1007/s40618-017-0651-9

**Published:** 2017-04-01

**Authors:** B. Wang, G. Sun, W. Qiao, Y. Liu, J. Qiao, W. Ye, H. Wang, X. Wang, R. Lindquist, Y. Wang, Y.-F. Xiao

**Affiliations:** 1 Crown Bioscience, Inc., Taicang, Jiangsu Province The People’s Republic of China; 2 Data Sciences International, St. Paul, MN USA

**Keywords:** Diabetes, Nonhuman primate, Continuous glucose monitoring, Implantable telemetry device, Glucose circadian

## Abstract

**Aims:**

Continuous blood glucose monitoring, especially long-term and remote, in diabetic patients or research is very challenging. Nonhuman primate (NHP) is an excellent model for metabolic research, because NHPs can naturally develop Type 2 diabetes mellitus (T2DM) similarly to humans. This study was to investigate blood glucose changes in conscious, moving-free cynomolgus monkeys (*Macaca fascicularis*) during circadian, meal, stress and drug exposure.

**Materials and methods:**

Blood glucose, body temperature and physical activities were continuously and simultaneously recorded by implanted HD-XG telemetry device for up to 10 weeks.

**Results and discussion:**

Blood glucose circadian changes in normoglycemic monkeys significantly differed from that in diabetic animals. Postprandial glucose increase was more obvious after afternoon feeding. Moving a monkey from its housing cage to monkey chair increased blood glucose by 30% in both normoglycemic and diabetic monkeys. Such increase in blood glucose declined to the pre-procedure level in 30 min in normoglycemic animals and >2 h in diabetic monkeys. Oral gavage procedure alone caused hyperglycemia in both normoglycemic and diabetic monkeys. Intravenous injection with the stress hormones, angiotensin II (2 μg/kg) or norepinephrine (0.4 μg/kg), also increased blood glucose level by 30%. The glucose levels measured by the telemetry system correlated significantly well with glucometer readings during glucose tolerance tests (ivGTT or oGTT), insulin tolerance test (ITT), graded glucose infusion (GGI) and clamp.

**Conclusion:**

Our data demonstrate that the real-time telemetry method is reliable for monitoring blood glucose remotely and continuously in conscious, stress-free, and moving-free NHPs with the advantages highly valuable to diabetes research and drug discovery.

## Introduction

Chronically dysfunctional carbohydrate metabolism results in diabetes due to a relative deficiency of insulin. Various animal models have been used in research for understanding diabetes and discovering novel therapies for the disease [1–4]. Nonhuman primates (NHPs) can naturally develop to Type 2 diabetes mellitus (T2DM) in a way similar to the progression and onset of T2DM in humans. Dysmetabolic NHPs have thus been used for diabetes and obesity research in many studies [5–8]. NHP models also play an important role in screening novel compounds for regulation of food intake, blood glucose, and/or body weight. The data from NHP models can result in discovery and validation of new mechanism or therapeutic strategy and target of dysmetabolic diseases [9, 10].

Using implanted telemetry device for continuous glucose monitoring has been very limited in biomedical research, especially in large animals. The conventional ways, such as handheld glucometer, clinical chemistry analyzer, or analox analyzer, are generally used for blood glucose measurement. These conventional methods require sampling blood periodically. Bleeding may induce stress and blood volume decline obviously in small animals if bleeding frequently. Periodical sampling may possibly miss some critical data points during sampling intervals. Thus, ‘around the clock’ real-time measurements of blood glucose in unstressed, moving-free animals have some unique advantages in pre-clinical research. In addition, sensing glucose from the blood directly has the potential to reduce or eliminate many issues commonly encountered with interstitial glucose sensing [11]. The HD-XG implantable glucose device (Data Sciences International, Saint Paul, MN, USA) provides continuous measurement of blood glucose, temperature, and locomotor activity for up to 2 months in rodents [12]. This study investigated the real-time changes of blood glucose during circadian, meal, stress procedures, and drug exposures by the modified HD-XG transmitter device in conscious, moving-free cynomolgus monkeys (*Macaca fascicularis*) with or without diabetes. An electrochemical glucose oxidase sensor placed in one of the monkey femoral arteries provided continuous real-time measurements of blood glucose. A temperature sensor contained in the implanted transmitter body provided continuous real-time temperature information. Signal strength variation due to physical activity of the subject provided a continuous real-time activity count as calculated in the DEM/MX2 (Data Sciences International, Saint Paul, MN, USA).

## Methods

### Animals and animal care

Experiments were performed in cynomolgus monkeys of either sex (Table 1). These monkeys were individually housed and maintained in our animal facility in accordance with the guidelines approved by the Association for Assessment and Accreditation of Laboratory Animal Care (AAALAC). The room temperature was maintained at ~21 °C with a 12-h light/dark cycle with lights off from 7 pm to 7 am. All the animals had access ad libitum to water and a complete, nutritionally balanced normal diet (Beijing Keao Xieli Feed Co., LTD, Beijing, China) enriched with seasonal fruit and vegetables. The experimental protocol was approved by the Institutional Animal Care and Use Committee (IACUC) of Crown Bioscience, Inc.

**Table 1:** Characteristics of the cynomolgus monkeys enrolled in the experiment

Group	Animal ID	Age (year)	Body weight (kg)	Fasting serum glucose (mg/dL)	Fasting serum insulin (mIU/mL)	HbA1c (%)
Normoglycemia	A07	5	4.7	58	5.3	4.3
	L02	8	6.1	53	49.6	5.5
	V01	9	7.8	63	29.9	4.4
Diabetes	B02	17	5.9	120	9.6	4.7
	J04	16	7.1	114	30.3	5.9

### Implantation of HD-XG telemetry device

**Fig. 1:**
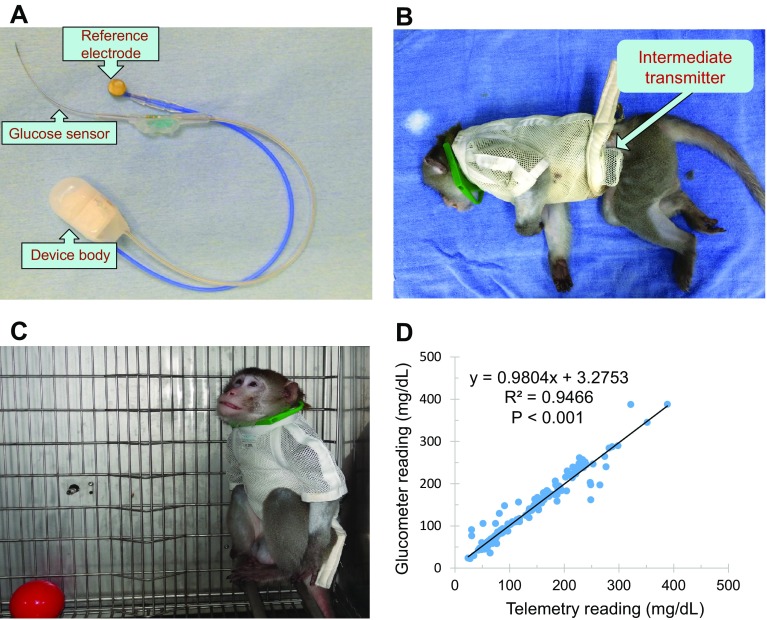
**a** Implantable HD-XG telemetry device for continuous monitoring of glucose, temperature, and locomotor activity. **b** Experimental monkey with an implantable device and wearing a monkey jacket in which a small transmitter was placed and used for signal collection from outside cage. **c** Signals of blood glucose, body temperature, and locomotor activity from a conscious, moving-free monkey were recorded continuously from its outside cage. **d** Significant correlation between the blood glucose levels measured by the telemetry method and glucometer test

An implantable telemetry device (HD-XG, DSI, St. Paul, MN, USA) consists of one glucose sensor lead, one reference lead and device body (Fig. 1a) and is packed and sterilized ready for use. The operation room and surgical tools were disinfected 1 day before surgical operation. On the experimental day for device implantation, each overnight-fasted monkey received ketamine (15 mg/kg, Fujian Gutian Pharmaceutical Co. Ltd., Fujian, China) and 0.01 mg/kg buprenorphine by intramuscular administration with additional ketamine (5 mg/kg) as needed during operation. Body temperature was monitored and maintained at ~37 °C by a thermostatically controlled warm water-circulating pad placed beneath the body. The skin around the intended incision site was shaved and made aseptic with betadine starting from the center of the incision region and spiraling outward. Then, the area was swept with a 70% ethanol gauze pad. The vital signs, such as heart rate, blood pressure, blood oxygen saturation, and respiration rate, were monitored during surgery. A small incision was made in the femoral area and one branch from the femoral artery was carefully dissected. The glucose sensor electrode of one HD-XG device was cannulated into the artery branch, and its tip reached the main femoral artery with ligation and fixation of the artery branch together with the sensor electrode. The reference electrode and device body were fixed subcutaneously in the place near by the femoral artery. The incision was then sutured and covered properly with gauze. The monkey was placed in a monkey jacket with an intermediate transmitter placed in jacket pocket (Fig. 1b, c). The monkey was returned to its housing cage after regaining consciousness. Buprenorphine at 0.01 mg/kg was injected intramuscularly every 6–12 h after implantation for 2 days and antibiotic amoxicillin at 7 mg/kg was also given intramuscularly if needed. The health of a surgical animal was closely monitored during 1 week recovery. Food and water were provided again after the surgical animal was returned to its housing cage and fully recovered from anesthesia.

### Device calibration

Fibrin and/or tissue adhered to the glucose sensor area of the electrode placed in the artery can affect glucose readings. To obtain an optimal performance and accuracy of glucose readings, the implanted HD-XG device must be calibrated by reference measurements of tail vein blood samples with the Nova StatStrip Glucose Meter (Nova^®^ Biomedical, Waltham, MA, USA) from time to time during study. Raw telemetry data are recorded in nanoamperes (nA) and calibration reference values are recorded in mg/dL. The calibration algorithm converts the telemetry (nA) data to the values that are equivalent to the appropriate mg/dL results.

#### Multi-point calibration

Multi-point calibrations were needed to establish a linear relationship between the sensor outputs and blood glucose levels at the beginning of initial data collection and also at the end of the study. During calibration, the blood glucose levels should differ by at least 100 mg/dL to minimize calibration errors due to the inaccuracy of glucose reference. Our study used intravenous injection of glucose to get multi-point calibrations.

#### Single-point calibration

Single-point calibrations were performed to prevent non-physiologic changes in the baseline glucose value over time during the study. Non-physiologic changes include enzyme instability due to sensor drifting or fibrin and tissue growth on the sensor electrode. Single-point calibrations were performed at least twice per week at the same time of day and during a time period when the animal’s blood glucose was relatively stable.

### Data collection

An implanted HD-XG glucose electrode continuously sensed the blood glucose and recorded the electrical signals during a study. After calibration with the glucose levels measured by the Nova StatStrip Glucose Meter (Nova^®^ Biomedical, Waltham, MA, USA), the recorded electrical signals were converted to glucose concentrations. In the meantime, blood glucose concentrations during various glucose tests or drug challenges were also measured by the Nova StatStrip Glucose Meter to further validate telemetry data if needed.

To test insulin resistance and β-cell insulin secretory response to acute hyperglycemia, intravenous glucose tolerance test (ivGTT) was conducted. The experimental animals were fasted overnight (around 16 h) and anesthetized with ketamine at 10 mg/kg (i.m.) initially plus additional dose (5 mg/kg, i.m.) during the procedure if needed. When blood glucose stabilized for approximately 30 min via observation of HD-XG glucose signals, the glucose solution (0.25 g/kg = 0.5 mL/kg of 50% dextrose) was intravenously infused via one cephalic vein within 30 s, and then the system was flushed with 5 mL heparinized saline to push in the residual glucose.

Hyperinsulinemic-euglycemic clamp was also tested in this study. The animals were fasted overnight (around 16 h) and anesthetized with ketamine at the initial dose of 10 mg/kg (i.m.) plus additional 5 mg/kg each time during the procedure if needed. In the period of narcosis, vital signs were monitored. The cephalic or saphenous veins were cannulated for glucose and insulin infusions. Insulin (biosynthetic human insulin, Novo Nordisk, Denmark) was diluted to 300 mIU/mL by isotonic saline to which 2 mL of the subject’s blood per 50 mL was added in order to avoid adhesion of insulin to the syringe plastic surface. After blood glucose stabilized for 30 min via observation of the HD-XG glucose signals, insulin infusion at various rates was given during the first 5 min to quickly adjust blood glucose near the targeted level (70 ± 5 mg/dL). However, diabetic monkeys needed 20–30 min for the adjustment. The infusion rate for the hyperinsulinemic-euglycemic clamp was then maintained at 40 mU/m^2^ surface area × min as reported previously [5, 13, 14]. A variable amount of 20% d-glucose was intravenously infused to maintain the blood glucose level. The initial glucose infusion rate (GIR) was at 5 or 6 mg/kg/min for normal monkeys and 2 mg/kg/min for diabetic monkeys, and then the infusion rate was adjusted until reaching a stable glucose level around 75 mg/dL for 30 min. During the weaning period, insulin infusion was terminated and blood glucose was continuously monitored with glucose infusion if needed until blood glucose was above 40 mg/dL and stable.

## Results

### Validation of the blood glucose levels measured by an implanted HD-XG device

To validate the reliability of the telemetry method, blood glucose levels were measured at different time points and with or without various challenges 1 week after device implantation. During the study, 187 blood glucose parameters were collected by both telemetry and glucometer methods in normoglycemic (*n* = 3) and diabetic (*n* = 2) monkeys. The blood glucose levels measured by the telemetry method were highly correlated with those read by the glucometer (Fig. 1d, Y = 0.9804*x* + 3.2753, *R*² = 0.9466, *p* < 0.001), which suggests that the telemetry method with the implantable HD-XG device is reliable for continuous blood glucose monitoring.

### Blood glucose fluctuations during daily activities

**Fig. 2:**
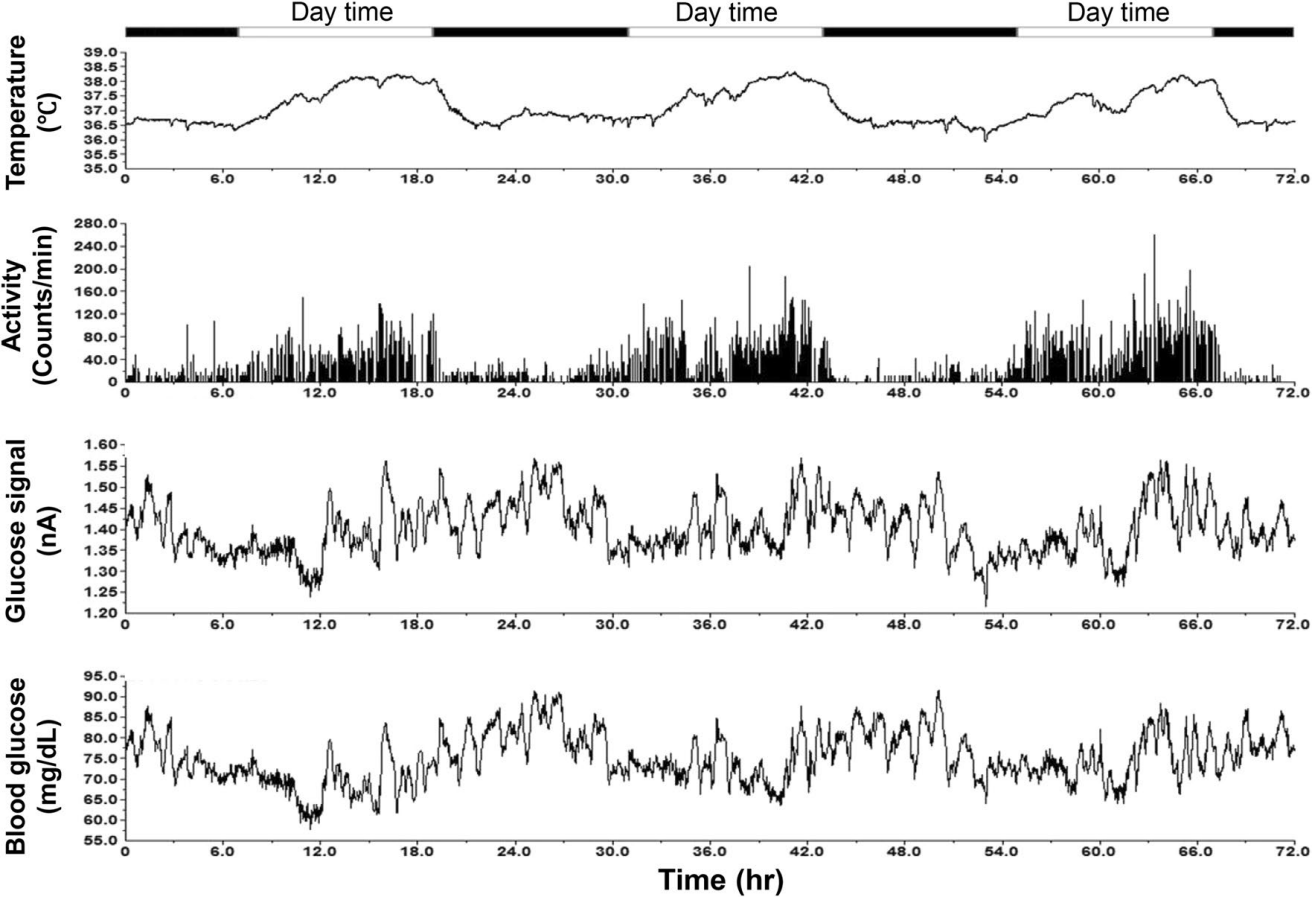
Representative traces collected continuously from a conscious monkey implanted with a HD-XG device. From *top* to *bottom*, body temperature (1st trace) with the maker on top to indicate day (*open bar*) and night (*black bar*) time, physical activity (2nd trace), blood glucose electrical signal (3rd trace), blood glucose level (4th trace) after calculation from the corresponding glucose electrical signal

**Fig. 3:**
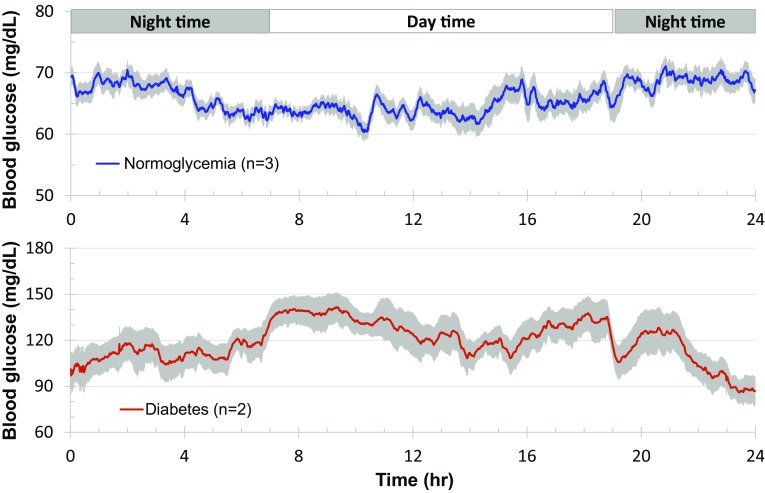
Daily blood glucose fluctuations averaged from 7-day consecutive recordings of each monkey in normal (*n* = 3, *upper panel*) and diabetic (*n* = 2, *low panel*) NHPs. The *solid line* in each panel represented the median level of instant blood glucose concentration from the seven consecutive days of each monkey for normoglycemia (*n* = 3) and diabetes (*n* = 2) ones, and the *shadowed areas* above or below the *line* were the deviations of high and low levels of blood glucose

**Fig. 4:**
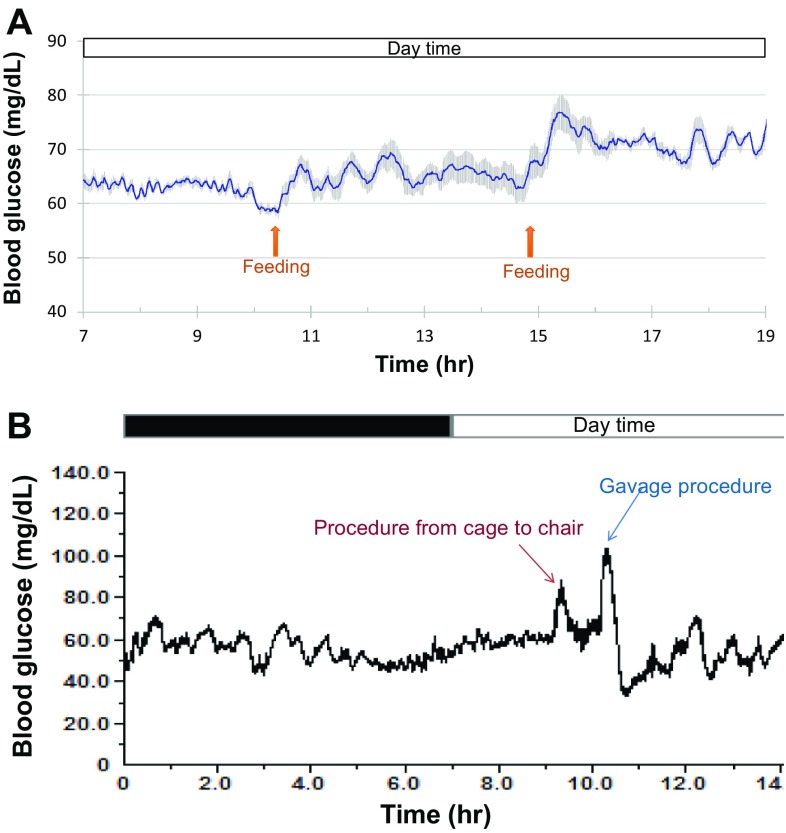
**a** Typical effects of feeding on blood glucose were shown from one conscious normoglycemia monkey. The *solid line* was the median level of instant blood glucose averaged from the seven consecutive days, and *shadowed areas* above or below the *line* were the deviations of high and low levels of blood glucose. **b** Representative stress responses to the operation procedures in one conscious normoglycemia monkey implanted with the telemetry device. Procedure from cage to chair, the monkey was removed from its cage and placed into a monkey chair. Gavage procedure, the monkey was chaired and encountered an oral gavage operation without any solution or drug delivery

With the clinical handheld glucometer method, continuously monitoring blood glucose day and night for a long term is impossible in conscious, stress-free, and freely moving monkeys. Figure 2 shows the circadian oscillations of blood glucose levels in a normoglycemic monkey for 3 days accompanied with the data from its physical activity and body temperature (Fig. 2). Such monitoring could last up to 10 weeks after one sensor was successfully placed in a femoral artery, and the HD-XG device body was implanted into one subcutaneous space. Interestingly, the pattern of blood glucose circadian in the normoglycemic monkeys (*n* = 3) differed from the one observed from the diabetes monkeys (*n* = 2). The averaged blood glucose level in the normoglycemic animals decreased gradually from 3 am to a low level within 2 h and then stayed at the low level until afternoon feeding at 3 pm (Fig. 3). However, the averaged blood glucose level in the diabetic animals increased gradually around 7 am to a new high level and then maintained at the relatively high level until late evening after 9 pm (Fig. 3). Both normoglycemic (*n* = 3) and diabetic (*n* = 2) animals enrolled in this study did not show obvious postprandial hyperglycemia after morning feeding, but the blood glucose level increased by 20 to 30% after afternoon feeding (Fig. 4a).

### Blood glucose response to experimental procedure or drug challenge

**Fig. 5:**
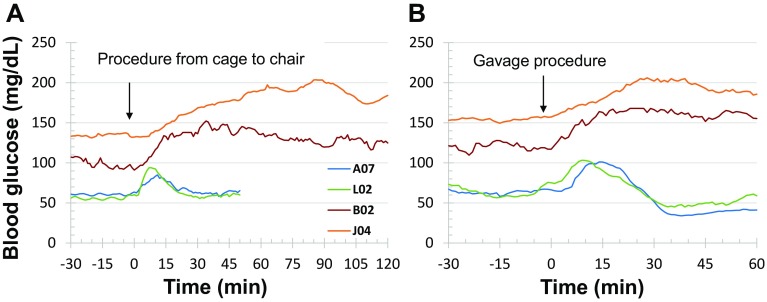
Effects of operation procedures on blood glucose in normoglycemia (*n* = 3) and diabetes (*n* = 2) monkeys with implanted telemetry device. Procedure from cage to chair (**a**), the monkeys were removed from their cages and placed into their monkey chairs. Gavage procedure (**b**), the monkeys were chaired and encountered oral gavage operation without delivery of any drug or solution. Compared with normoglycemic monkeys, the stress responses to the operation procedures were markedly prolonged in diabetes animals

To examine if an experimental procedure could cause stress and alter blood glucose level, an overnight-fasted monkey was moved out of its cage by grabbing his neck collar to let him sit in a monkey chair. This procedure increased the blood glucose level approximately 30 mg/dL, which lasted about 10–20 min (Figs. 4b, 5a) in normoglycemic monkeys (*n* = 3). However, the same procedure increased blood glucose level by 50–70 mg/dL in the diabetic animals (*n* = 2), which lasted over 2 h (Figs. 3, 5a).

Oral or nasal gavage is a conventional procedure frequently used for drug dosing in NHPs. To test if such procedure could also cause stress and alter blood glucose levels, an overnight-fasted monkey placed in a monkey chair underwent a sham oral gavage procedure (placing a gavage tube into the stomach without giving food, drug, or solution). The procedure alone caused the increase in blood glucose levels by 30–50% in both normoglycemia and diabetes monkeys (Figs. 4b, 5b). However, the procedure-induced hyperglycemia lasted much longer in diabetes (>60 min, *n* = 2) than in normoglycemia (about 20 min, *n* = 3) monkeys (Figs. 4b, 5b).

**Fig. 6:**
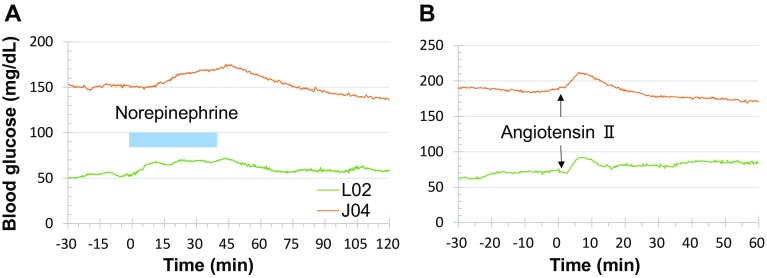
Effects of norepinephrine and angiotensin II on blood glucose in normoglycemic and diabetic monkeys implanted with the telemetry device. *Left panel* norepinephrine at 0.4 µg/kg/min was intravenously infused for 40 min in anesthetized (10 mg/kg ketamine, intramuscularly) normoglycemic (L02) and diabetic (J04) monkeys. The *blue bar* represents the time period with norepinephrine infusion. *Right panel* angiotensin II at 2 µg/kg was intravenously injected in conscious normoglycemic (L02) and diabetic (J04) monkeys. The *arrows* indicated the time point for angiotensin II bolus injection. L02 and J04 were the identification codes of the normoglycemic and diabetic monkeys used in both *left* and *right panels*

Stress-induced hyperglycemia during chairing or gavage procedure most likely resulted from neuronal alert and hormonal secretion. To test if stress hormone could cause hyperglycemia, norepinephrine and angiotensin II were administered intravenously to the monkeys with implanted HD-XG devices. The animals with norepinephrine challenge were fasted overnight (around 16 h) and anesthetized with ketamine 10 mg/kg (i.m.) initially and additional 5 mg/kg if needed during the procedure. After the blood glucose level was stable for 30 min, norepinephrine solution at 0.4 μg/kg/min was infused intravenously for 40 min. Blood glucose gradually increased and reached the peak near the end of infusion in both normoglycemic (L02) and diabetic (J04) monkeys (Fig. 6a). The hyperglycemic response to norepinephrine slowly subsided after cessation of intravenous norepinephrine infusion. Interestingly, the blood glucose level was lower at the end of infusion than the pre-norepinephrine level in the diabetic animal (J04, Fig. 6a).

The overnight-fasted monkeys were restrained in the monkey chair. After stabilization of their blood glucose levels based on the HD-XG glucose signals, angiotensin II at 2 μg/kg was administered intravenously by bolus injection. Blood glucose level gradually increased and reached the peak within 10 min after angiotensin II injection in both normoglycemic (L02) and diabetic (J04) monkeys (Fig. 6b). The hyperglycemic response to angiotensin II slowly waned and lately even went lower than the pre-angiotensin II level in the diabetic animal (J04, Fig. 6b). It is interesting that intravenous bolus injection of acetylcholine at 1 μg/kg did not alter blood glucose levels in both normoglycemic and diabetic monkeys implanted with HD-XG telemetry devices (data not shown).

### Glucose tolerance test and other tests in NHPs with implanted HD-XG device

To evaluate β-cell function, ivGTT, oGTT, ITT, GGI, and glucose clamp were performed in the experimental monkeys according to the method reported previously [5, 6, 14–17]. The results obtained from implanted HD-XG telemetry device were compared with those measured by the glucometer (StatStrip Xpress meter, Waltham, MA, USA) via the tail prick method.

#### ivGTT

**Fig. 7:**
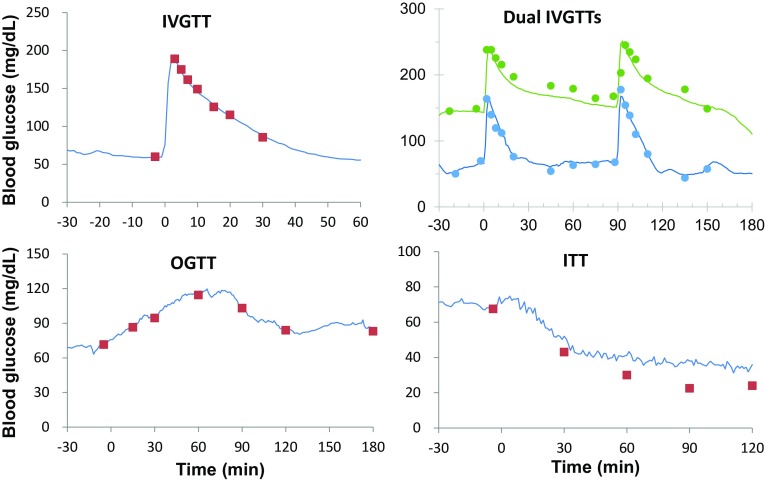
Representative data to make comparison of the outcomes measured by glucometer and telemetry. Blood glucose levels responded to single ivGTT in one normoglycemic animal (*upper left*) and dual ivGTTs in one normoglycemic (*Blue*) and one diabetes (*Green*) monkeys (*upper right*) measured with both telemetry (*solid line*) or glucometer (*scatted squares* or *circles*). Blood glucose levels responded to single oGTT (*low left*) and ITT (*low right*) in one normoglycemic monkey were measured with both the telemetry (*solid line*) or glucometer (*scatted squares*) methods

After intravenous glucose injection blood glucose changes were monitored and recorded by the implanted telemetry device (Fig. 7, upper panels). Also, blood glucose levels were measured by the glucometer (StatStrip Xpress meter) via the tail prick method immediately before and at 3, 5, 7, 10, 15, 20, and 30 min after glucose infusion for single ivGTT (Fig. 7, IVGTT) or longer according to the observation from HD-XG glucose signals for dual ivGTTs (Fig. 7, Dual IVGTT). The results showed that the glucose levels measured by telemetry matched well to the readings by glucometer.

#### oGTT

The experimental animals (normoglycemia, *n* = 3 and diabetes, *n* = 2) were fasted overnight (approximately 16 h) and restrained in the monkey chair. When blood glucose stabilized for around 30 min via observation of the HD-XG glucose signals, the glucose solution (1.75 g/kg = 5 mL/kg of 35% dextrose) was given to the animal via oral gavage. Blood glucose was measured by glucometer (StatStrip Xpress meter) immediately before and at 15, 30, 60, 90, 120, and 180 min after glucose administration. Meanwhile, blood glucose levels were monitored by the implanted HD-XG device throughout the entire test. The results were plotted and correlated with each other very well (Fig. 7, OGTT).

#### ITT

ITT is used to examine if the body produces enough ACTH (adreno-cortico-trophic hormone) and growth hormone under stress. The ‘stress’ in this test was to lower blood sugar (hypoglycemia) by injected insulin under very controlled conditions. ACTH stimulates the adrenal glands to make cortisol which is a steroid hormone and has many functions, including balancing the effect of insulin in regulating blood glucose levels. Five animals (normoglycemia, *n* = 3 and diabetes, *n* = 2) were fasted overnight (16 h) and restrained in the monkey chair. When blood glucose levels stabilized for around 30 min via observation of the HD-XG glucose signals, the insulin solution (0.15 U/kg = 0.15 mL/kg of insulin injection) was given to each animal via S.C. injection [5]. Blood glucose levels were measured by the glucometer method (StatStrip Xpress meter) immediately before and at 30, 60, 90, and 120 min after insulin administration (Fig. 7, ITT). Meanwhile, blood glucose levels were monitored and recorded by implanted HD-XG devices. The results were plotted against the time and were correlated very well between the telemetry results and the glucometer readings.

#### GGI

**Fig. 8:**
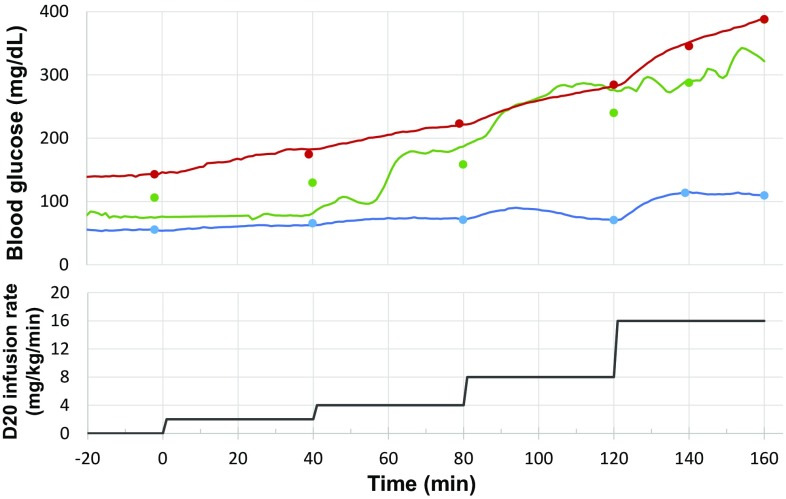
Comparison of the outcomes measured by glucometer and telemetry. Graded glucose infusion in anesthetized normoglycemic (*blue line, n* = 1) and diabetic (*green* and *red lines*, one animal per *line*) monkeys with similar glucose levels measured by telemetry (*solid line*) and glucometer (*solid dotted circles*). Each animal was anesthetized with ketamine at 10 mg/kg (i.m.) plus additional dose 5 mg/kg each time during procedure if needed. The *upper panel* shows the glucose levels and *low panel* shows the graded glucose infusion rates

The animals were fasted overnight (around 16 h) and anesthetized with an initial dose of ketamine at 10 mg/kg (i.m.) plus additional dose at 5 mg/kg each time during the procedure if needed. Then, an intravenous catheter was placed in one cephalic or saphenous vein. After blood glucose stabilized for 30 min via observation of the HD-XG glucose signals, glucose (20% dextrose) was continuously infused via the cannulated vein for 40 min per stage with the initial 2 mg/kg/min and then the dose was doubled at each successive stage up to 16 mg/kg/min by four stages (Fig. 8, low panel). Blood glucose was monitored and recorded by the implanted telemetry device and also measured by the glucometer (StatStrip Xpress meter) with the tail prick method at the time points immediately before and 40, 80, 120, and 160 min after glucose infusion. The blood glucose levels measured by the telemetry system matched well with those tested by the glucometer method (Fig. 8).

#### Hyperinsulinemic-euglycemic clamp

**Fig. 9:**
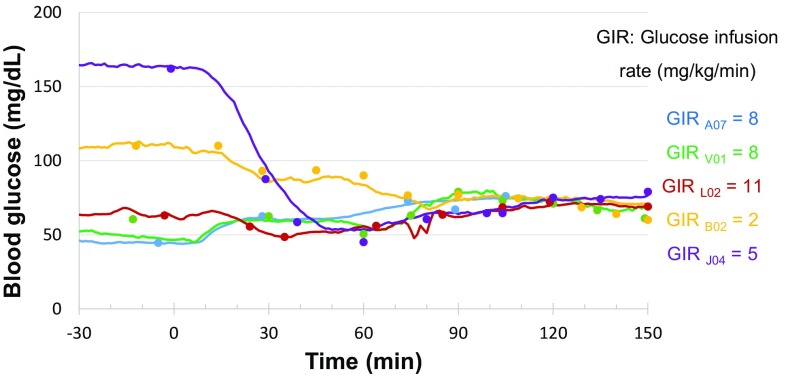
Comparison of the blood glucose levels measured by glucometer (*scatted dots*) and telemetry (*solid lines*) during glucose clamp. Hyperinsulinemic-euglycemic clamps were conducted in normoglycemic (*n* = 3) and diabetic (*n* = 2) monkeys. Blood glucose was measured by both telemetry (*solid line*) and glucometer (*solid scatted circles*) methods. The animals were anesthetized with ketamine at 10 mg/kg (i.m.) initially and then 5 mg/kg each time during clamp procedure if needed

The blood glucose levels measured by the HD-XG telemetry method matched the glucometer readings well (Fig. 9). Such match was observed with the glucose levels not only above 100 mg/dL or even above 150 mg/dL (Fig. 9, animal B02 and J04), but also low approximately 50 mg/dL (Fig. 9, animal A07, V01, L02). These results indicate that HD-XG telemetry technology can be used for glucose clamp, the golden standard method in diabetes research.

## Discussion

This is the first successful demonstration of telemetry monitoring of blood glucose continuously in NHPs. Our data show that blood glucose in conscious, non-stressed cynomolgus monkeys can be accurately measured and recorded by the implanted HD-XG glucose sensor and its data collection system. The method can monitor and record instant blood glucose level, body temperature, and animal activity continuously for up to 10 weeks. The blood glucose parameters collected by the telemetry method correlated significantly well with the readings tested by the classical glucometer method at different glucose levels (Fig. 1d), even under various challenges (Figs. 7, 8, 9). The advantages of glucose monitoring by the implantable telemetry device in NHPs include: (1) long-term monitoring; (2) 24 hr consecutive data collection to observe circadian and postprandial glucose changes; (3) no bleeding during measurement; (4) no restriction even in fully conscious, free-moving animals; (5) no anesthesia even for ivGTT or glucose clamp; (6) no stress as each animal with implanted device moves freely; (7) less labor intensive procedure during ivGTT, oGTT, ITT or clamp; and (8) instant glucose readings without blood test. Our study demonstrates that remotely and continuously monitoring blood glucose via implanted HD-XG telemetry device in conscious, stress-free, and freely moving monkeys is feasible and provides a sophisticated approach to investigate glucose changes due to daily activities, neuronal and hormonal changes, or physical/chemical challenges. In fact, moving a monkey from its cage to the monkey chair or proceeding an oral gavage induced hyperglycemia in both normoglycemia or diabetes monkeys (Fig. 5). Such stress-induced hyperglycemia could be simulated with intravenous administration of stress hormones (Fig. 6). If glucose tests with glucometer or blood sampling are infrequent, such procedure-induced hyperglycemia in conscious animals could be missed or be misinterpreted as a baseline or treatment effect. Therefore, continuous monitoring of blood glucose by the implantable telemetry device could avoid not only some unnecessary stressful procedures but also some misinterpretation of data.

It is interesting that compared with nighttime, blood glucose was relatively lower during most daytime in the normoglycemic monkeys, but not the case in the diabetic ones (Fig. 3). The daily circadian of blood glucose in the diabetes monkeys was higher during daytime with starting from very early morning (Fig. 3). This phenomenon could result from insulin resistance and relative insulin deficiency in diabetic NHPs, which is similar to the observation in diabetic patients [18]. Clinically, glucose circadian changes are influenced by levels of both insulin and the counter-regulatory hormones, such as glucagon, adrenaline, growth hormone, and cortisol. There is a surge in the amount of released growth hormone beginning from the middle night, followed by a surge in cortisol to increase liver blood glucose production. These processes are offset by increased insulin secretion to maintain blood glucose relatively stable in normoglycemic individuals. However, in diabetic patients, hormone changes during sleep can have a powerful effect on morning blood glucose level, an event dubbed the “dawn phenomenon” [19, 20].

Our data clearly showed that physical activity and body temperature gradually declined from 7 pm each day (Fig. 2). Then, physical activity and body temperature changes began to increase from very early morning (Fig. 2). Under normal physiological condition, our body would enhance glucose generation and usage according to hormone secretion, physical activity, and energy needs. It is well recognized that the main cause of T2DM is a combination of varying degrees of insulin resistance and relative insulin deficiency [21–23]. Due to insulin resistance and impaired glucose handling (Fig. 5), stress or experimental procedure resulted in hyperglycemia which took much longer time to decline to the pre-level in diabetic monkeys than in normoglycemic ones (Fig. 5). Therefore, the increased activities from early morning to later in the day resulted in the increase in blood glucose and less physical activity after 7 pm led to gradual decrease in blood glucose in diabetes NHPs due to their impaired glucose handling (Fig. 3). However, the daily circadian of blood glucose in the normoglycemic NHPs was opposite to that in diabetes animals (Fig. 2). The increase in physical activity resulted in low blood glucose during early morning and most of other daytimes, except the time after afternoon feeding. Less physical activity after 7 pm showed resulted in higher blood glucose until early morning (Figs. 2, 3). The potential explanation is that glucose handling (M rate) was much better in normoglycemic monkeys than in diabetic ones [5]. As physical activity enhances insulin secretion and sensitivity in normoglycemic animals [24, 25], the body can regulate more glucose per unit time and decreased blood glucose during physical activity. Therefore, the pattern of glucose circadian in diabetic monkeys differs from that in normoglycemic NHPs. This novel finding results from continuous glucose monitoring by the implantable telemetry method which provides the instant blood glucose concentration 24 h per day for up to 10 weeks (Figs. 2, 3). It would be very difficult to find such circadian difference between normoglycemia and diabetes in NHPs if the classical glucometer method was used for periodical determination of blood glucose, especially during night. Also, the glucometer method would obviously disturb the monkey’s normal activity cycle a lot which may obscure the true pattern of blood glucose fluctuation if frequent measurements are made.

The monkeys housed in our animal facility were fed twice per day. The postprandial increase in blood glucose was very minor with a few small fluctuations after morning feeding and was more obvious with one main peak after afternoon feeding (Fig. 4a). This pattern of food intake in NHPs differs from humans. Generally speaking, humans complete their meals in a certain period of time (most likely less than 1 h). Blood glucose increase after a meal is more obvious and relatively predictable in humans. However, most of our housed monkeys might not complete their meals in a relatively short period of time, especially after morning feeding. The monkeys could eat and play with their food for quite a while, which initiated those small fluctuations of blood glucose after morning feeding (Fig. 4a). Postprandial increase in blood glucose was more obvious with one main peak after afternoon feeding. This peak potentially indicated the change of the housed monkey behavior. They were smart to intake enough food before 7 pm as the access became difficult after light off. This postprandial blood glucose pattern in housed NHPs also resulted from the advantage of the implantable telemetry device which did not affect animal normal behavior.

Clinically, the FDA-approved Continuous Glucose Monitoring (CGM) system (Dexcom G4 PLATINUM Continuous Glucose Monitoring System, Dexcom, Inc., San Diego, CA, USA) can sense interstitial glucose frequently in diabetic patients to inform exogenous insulin delivery timing and dosage. Compared with the arterial glucose telemetry monitoring method used in this study, the advantages of the clinical CGM system (subcutaneously) are relatively easier installation, less expensive, less invasive, and more acceptable safety profile which becomes the mainstay for patient use. For animal research, the disadvantages of the clinic CGM system can firstly manifest moderately delayed glucose readings (5–12 min) [26–29] and likely worsen with implantation time as encapsulation develops [30, 31]. Secondly, the CGM system readings of glucose level can vary due to local fluctuations of subcutaneous blood flow by the change of temperature and/or mechanical pressure [32, 33]. Thirdly, subcutaneously inflammatory response results in biofouling and encapsulation, which limits sensor service time (less than 2 weeks) [26, 34]. Fourthly, fixation of subcutaneously inserted sensor for stable data collection in physically active monkeys is very challenging. To stabilize implanted sensor and to avoid any delay of glucose reading, this study used the implanted HD-XG telemetry device to access arterial vasculature and provided real-time blood glucose information [12].

In summary, the HD-XG telemetry method was successfully used to continuously monitor and record real-time blood glucose for the first time in conscious, freely moving NHPs. This novel technology allows researchers to discover blood glucose circadian and changes due to physical activities or natural behaviors, such as eating and sleeping, as well as stress or drug challenges. By this telemetry approach, we can now continuously collect blood glucose data for up to 10 weeks without blood sampling which can cause animal stress and test variability commonly associated with classical glucose assays (glucometer or lab blood test). Therefore, using such telemetry technology can help us to further understand glucose metabolism under more natural physiologic condition.

## Abbreviations

AAALAC: Association for Assessment and Accreditation of Laboratory Animal Care
CGM: Continuous glucose monitoring
GIR: Glucose infusion rate
IACUC: Institutional Animal Care and Use Committee
ITT: Insulin tolerance test
GGI: Graded glucose infusion
ivGTT: Intravenous glucose tolerance test
NHPs: Nonhuman primates
oGTT: Oral glucose tolerance test
T2DM: Type II diabetes mellitus
